# Reversal of Epithelial–Mesenchymal Transition by Natural Anti-Inflammatory and Pro-Resolving Lipids

**DOI:** 10.3390/cancers11121841

**Published:** 2019-11-21

**Authors:** Chang Hoon Lee

**Affiliations:** College of Pharmacy, Dongguk University, Seoul 100-715, Korea; uatheone@dongguk.edu; Tel.: +82-109-755-1746

**Keywords:** epithelial mesenchymal transition, inflammation, malignant cancer, natural anti-inflammatory compounds, pro-resolving lipids

## Abstract

Epithelial mesenchymal transition (EMT) is a key process in the progression of malignant cancer. Therefore, blocking the EMT can be a critical fast track for the development of anticancer drugs. In this paper, we update recent research output of EMT and we explore suppression of EMT by natural anti-inflammatory compounds and pro-resolving lipids.

## 1. Introduction

The epithelial–mesenchymal transition (EMT) is defined as a phenomenon that epithelial cells transform into mesenchymal cells [1]. EMT plays a key role in cancer progression and fibrosis. Many researchers and pharmaceutical companies have tried to develop novel EMT blockers due to its importance in such diseases [2,3,4].

Inflammation is one of ten in cancer hallmark [5]. It is a critical factor of tumor microenvironment affecting EMT. Several reviews have emphasized role of inflammation in EMT [6]. However, there are a few reviews that deal with the blocking of EMT by natural anti-inflammatory compounds and pro-resolving lipids.

In this review, I have dealt with ingredients derived from natural products that were not covered in the 2018 review on EMT [6]. I have also added the story of inflammasomes, which play an essential role in the early steps of inflammation, and how they are involved in EMT. Naturally derived compounds that control these inflammasome-related molecules in the EMT have been discussed. About the resolution of inflammation, the newly discovered pro-resolving lipids including RvTs are added and the receptors specifically acting on pro-resolving lipids have also been discussed. Understanding the action of natural anti-inflammatory compounds and pro-resolving lipids with anti-EMT activities might provide a new armory to suppress the progression of cancer.

## 2. EMT in Cancers

**Figure 1:**
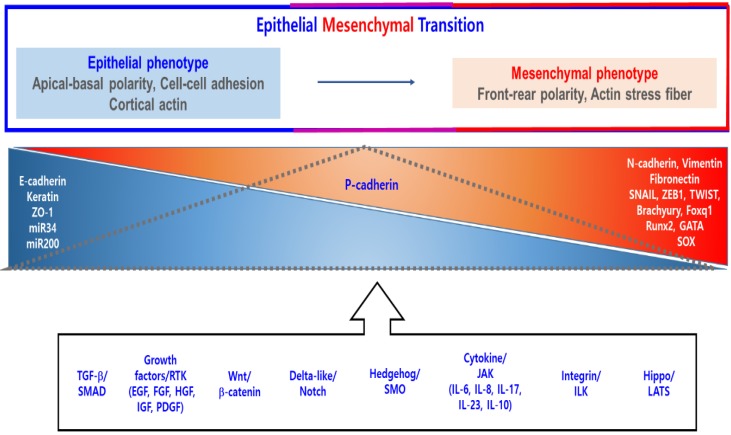
Epithelial–mesenchymal transition (EMT).E-cadherin: Epithelial cadherin; EGF: Epidermal Growth Factor; FGF: Fibroblast Growth Factor; GATA: GATA Binding Protein; HGF: Hepatocyte Growth Factor; IGF: Insulin Like Growth Factor; ILK: Integrin Linked Kinase; JAK: Janus Kinase; LATS: Large Tumor Suppressor Kinase; miR34: microRNA 34; miR200: microRNA 200; N-cadherin: Neural cadherin; P-cadherin: Placental cadherin; PDGF: Platelet Derived Growth Factor; Runx2: RUNX Family Transcription Factor 2; SMAD: Sma- And Mad-Related Protein; SMO: Smoothened, Frizzled Class Receptor; SOX: SRY-Box Transcription Factor; TGF-β: Transforming Growth Factor Beta; Wnt: Wingless-Type MMTV Integration Site Family; ZEB1: Zinc Finger E-Box Binding Homeobox 1; ZO-1: Zona Occludens 1.

EMT shows the reduced expression of epithelial markers including E-cadherin and keratins and the increased expression of mesenchymal marker proteins such as vimentin and N-cadherin via actions of transcription factors including as SNAIL1 and ZEB1 (Figure 1) [6]. The molecular mechanism of EMT process has been well explained in the report (references in it) [1]. In this part, we will briefly update the concept that reflects recent achievements for EMT.

EMT occurs by a various mediators from tumor microenvironments via receptor through signal transduction. EMT-related transcription factors blocks the expression of epithelial cell-marker genes and evoke mesenchymal-marker genes. E-cadherin, keratin, ZO-1, miR-34, and miR-200 belong to the epithelial markers, and N-cadherin, vimentin, fibronectin, SNAIL, ZEB1, TWIST, Brachyury, Foxq1, Runx2, GATA, and SOX belong to the mesenchymal markers. P-cadherin is the marker of partial EMT. The dot triangle indicates the relative ratio of p-cadherin expression. Modified from Lee’s report [6].

### 2.1. Adaptation to New Concepts of EMT

#### 2.1.1. Signaling Pathways in the EMT Process

A typical signaling pathway of EMT is the transforming growth factor-β1 (TGF-β1) pathway. TGF-β1 induces EMT via SMAD-dependent or non-SMAD signaling pathway [7]. Growth factors including FGF, HGF, IGF1, EGF, and PDGF via receptor tyrosine kinase can induce EMT via signaling pathway of PI3K-AKT and ERK MAPK [8,9,10,11]. Wnt signaling, hedgehog signaling, Notch signaling, hypoxia, and inflammatory tumor microenvironment also involves in EMT [5]. Recently, it has been shown that hippo signaling is also involved in EMT [12]. YAP and TAZ can enhance EMT through upregulation of EMT transcription factors such as forkhead box C2 (FOXC2), snail family zinc finger 1/2 (SNAIL1, SLUG), twist-related protein 1 (TWIST1), and ZEB1 [12,13,14,15].

#### 2.1.2. Transcription Factors Involved in EMT

Novel players are newly recognized as regulatory transcription factor in the EMT. Brachyury, the T-box transcription factor, is a novel transcription factor implicated in the EMT of cancer cells [16]. Brachyury is known as the target gene of WNT, one of the major signaling pathways of EMT [17]. Foxq1, one of forkhead transcription factor, has also regarded as a novel transcription factor mediating the EMT of gastric cancer [6,18]. Runt-related transcription factor 2 (Runx2) belongs to the runt-related transcription factor family [19]. Runx2 plays a key role in EMT of hepatocellular carcinoma (HCC) [20]. GATA transcription factors are also implicated in the EMT of cancer cells [21]. Serine 161 and serine 187 phosphorylated GATA1 by PAK5 can promote EMT of breast cancer cells by recruiting histone deacetylase 3/4 to E-cadherin promoter [22]. Other players of EMT include SRY-box (SOX) transcription factors [23]. Sox4 acts as a master regulator in EMT of cancerous breast epithelial cells [24].

#### 2.1.3. Partial EMT

EMT could be not defined as a dichotomous transition from epithelial status to mesenchymal one of cells [6,25].

Partial EMT with both epithelial and mesenchymal cell markers was proposed (Figure 1) [25]. Cells that have undergone with partial EMT have the capability showing collective sheet or cluster migration [26]. Partial EMTed cells have the competitiveness in that not all cells need to respond to EMT signals. Thus, they can far more efficiently execute plasticity in converting to a colonization state of metastasis via MET [25].

#### 2.1.4. Parallelism between Cancer Stem Cell and EMT

A cancer stem cell (CSC) is a cancer cell having the ability of self-renewal and differentiation. It divides to progenitor cancer cells. It is a culprit of cancer recurrence and metastasis [27]. Several EMT transcription factors and inducers can evoke the expression of cancer stem cells markers, thereby enhancing the capability to initiate cancer, a typical characteristic of cancer stem cells [28]. TGF-β promotes the de-differentiation of human basal breast non-CSCs into CSCs via ZEB1, suggesting that the activation of EMT in cancer cells by TGF-β/ZEB1 is closely linked to the de-differentiation of cancer cells into the CSC state [29].

### 2.2. Focus on Chemoresistance and Immune Evasion of EMT in Cancers

In the course of EMT, epithelial cancer cells can lose contacts between cells and apicobasal polarity but gain enhanced migration and invasion [1,6]. EMT has also strong influences on several hallmarks of cancer including cancer initiation, immune evasion, proliferation, survival, and resistance to therapeutics [6,30]. We will briefly update EMT’s roles in cancer such as chemoresistance and immune evasion.

#### 2.2.1. Chemoresistance

Chemoresistance to anticancer chemotherapeutics implies that cancer cells can survive despite the administration of an anticancer drug in a dose that can usually kill cancer cells. A significant correlation has been found between EMT-related gene expression and chemo-resistance to anticancer therapy [6,30,31]. Although the role of EMT in metastasis is disputable by some group, EMT is crucial for anticancer drug resistance [32,33]. For example, TWIST-mediated EMT is related to sorafenib resistance to advanced HCC [34]. Therefore, EMT should be understood as a predictor of chemoresistance for anticancer drugs.

#### 2.2.2. Immune Evasion

EMT has been understood as a key mechanism of immune escape of cancer cells. When snail1, an EMT transcription factor, is ectopically expressed in MCF7 breast cancer cells, cancer cell lysis executed by TNF-α-induced CTL is curtailed [35]. When snail1 is overexpressed in B16 melanoma cells, CTL-induced lysis is reduced and maturation of dendritic cells is inhibited while inhibitory Treg-like CD4^+^ Foxp3^+^ cells are expanded [36]. 

Enhanced EMT properties in cancer cells (A549, MCF7, and HepG2) by the TGF-β, IFN-γ, and TNF-α may affect differentiation and death of natural killer (NK), T, and B cells [37]. Reduced miR-200 and enhanced ZEB1 expression in lung cancer cells not only can evoke EMT, but also can lead to enhanced expression of PD-L1, which is related to the exhaustion of CD8^+^ T lymphocytes in lung cancer tissues [38]. In contrast, activated CD8^+^ T cells provoke mammary epithelial tumor cells to experience EMT, thus obtaining cancer-initiating power of breast cancer stem cells [6,39].

## 3. Induction of EMT by Mediators from the Chronic Inflammatory Tumor Microenvironment

### 3.1. Upgrade of Inflammation Concept: From Initiation (alpha) to Resolution (omega)

Inflammation is intrinsically a protective process via microcirculation. Local or systemic inflammatory reactions delete the causing stimuli and reboot repair and healing processes of tissue [40].

Acute inflammation has two phases: initiation (alpha) and resolution (omega). Inflammation starts by the soluble inflammatory mediators such as complement, cytokines including chemokines, free radicals, vasoactive amines, and eicosanoids (including prostaglandins) by adjacent cells of the infected or injured part in the body [6,40,41].

Inflammasome is a multiprotein oligomer responsible for the activation of inflammatory responses and consists of NLRP protein such as NLRP3, ASC, and procaspase-1 [42,43]. The inflammasome can promote the maturation and secretion of interleukin 1β (IL-1β) and IL-18 [44].

Inflammasomes are involved in the EMT of cancer or other epithelial cells. For example, knockdown of NLRP3 alleviates high glucose or TGF-β1-induced EMT in human renal tubular cells [45]. NLRP3 regulates cellular proliferation and metastasis via EMT and the PTEN/AKT signaling pathway [46]. NLRP3 inhibition can attenuate silica-induced EMT in human bronchial epithelial cells [47]. NLRP3 also participates in the regulation of EMT in bleomycin-induced pulmonary fibrosis [48]. Uric acid can activate NLRP3 inflammasome in the EMT in the kidney of rats [49]. NLRP3 appears to be important for EMT since inflammasome-independent NLRP3 is enough to EMT in colon cancer cells [50].

**Figure 2:**
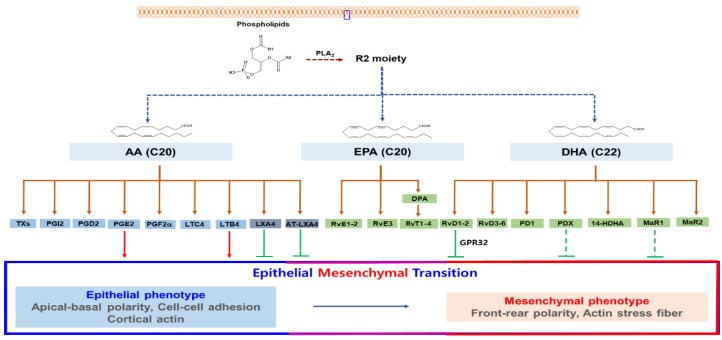
Inflammatory contribution to EMT. PGE2 and LTB4 produced from arachidonic acid can induce EMT in epithelial cancer cells. In contrast, LxA4 and AT-LxA4 from arachidonic acid can repress EMT in cancer cells. RvD1, RvD2, PDX, and MaR1 from DHA can suppress EMT in cancer cells or immortalized cells. AT-LxA4 (15(R)-lipoxin A4) is produced from 15(R)-HETE from arachidonic acid by aspirin trigger. Modified from Lee’s report [6]. 14-HDHA: 14-hydroxy Docosahexaenoic Acid; AA: arachidonic acid; AT-LxA4: Aspirin-triggered lipoxin A4; EPA: Eicosapentaenoic acid; DHA: Docosahexaenoic acid; DPA: Docosapentaenoic acid; MaR1: Maresin 1; MaR2: Maresin 2; PD1: Protectin 1; PDX: Protectin X; PGD2: Prostaglandin D2; PGF2a: ProstaglandinF2a; PGI2: Prostaglandin I2; PLA2:Phospholipase A2; LTB4: Leukotriene B4; LTC4: Leukotriene C4; LxA4: LIpoxin A4; RvD1-2: Resolvin D1-2; RvD3-6: Resolvin D3-6; RvE1-2: Resolvin E1-E2; RvE3: Resolvin E3; RvT1-4: 13-series resolvins; TXs: Thromboxane.

The resolution (omega) phase of inflammation releases specialized lipid mediators that can actively prevent further progress of inflammation and enhance resolution of inflammation [51]. A new specialized group of lipids that can actively terminate inflammation has been found by Serhan et al. [52]. These kinds of lipids include lipoxins (Lx), resolvins (Rvs), protectins (PDs), and maresins (MaRs; Figure 2). They exhibit inflammation-suppressing action with pro-resolving effect that can promote efferocytosis [41,52].

13-series resolvins (RvT1-4) derived from docosapentaenoic acid (DPA) have been newly discovered [53]. Briefly describing their production, human platelets pre-treated with aspirin or atorvastatin convert omega-3 DPA (DPAn-3) to a 13S-hydroperoxy intermediate via aspirin-treated or atorvastatin-treated COX-2. This intermediate is converted into four RvTs via ALOX5 enzyme activity exerted on the nearby neutrophils [53]: RvT1 (7,13R,20-trihydroxy-DPAn-3); RvT2 (7,8,13R-trihydroxy-DPAn-3); RvT3 (7,12,13R-trihydroxy-8Z,10E,14E,16Z,19Z-DPAn-3); and RvT4 (7,13R-dihydroxy-DPAn-3). Four RvTs are also formed by a mixture of human neutrophils and vascular endothelium cells, which are found in infected rodent and human tissues. [52].

### 3.2. EMT Inducers from Chronic Inflammatory Tumor Microenvironments

The tumor microenvironment mainly influences the progression of cancers via secretion of various factors that cause EMT [54,55]. Cancer-related chronic inflammation is described as a chaotic state where both pro-inflammatory and anti-inflammatory signals are present to permit tumor growth and immune evasion [6]. Besides, the tumor microenvironment contributes to the cancer heterogeneity. Therefore, the tumor microenvironment has been regarded as a promising target for the cure of cancer. Thus, Vanneman and Dranoff have demonstrated a novel way of curing cancer by re-educating the tumor microenvironment [56].

**Figure 3:**
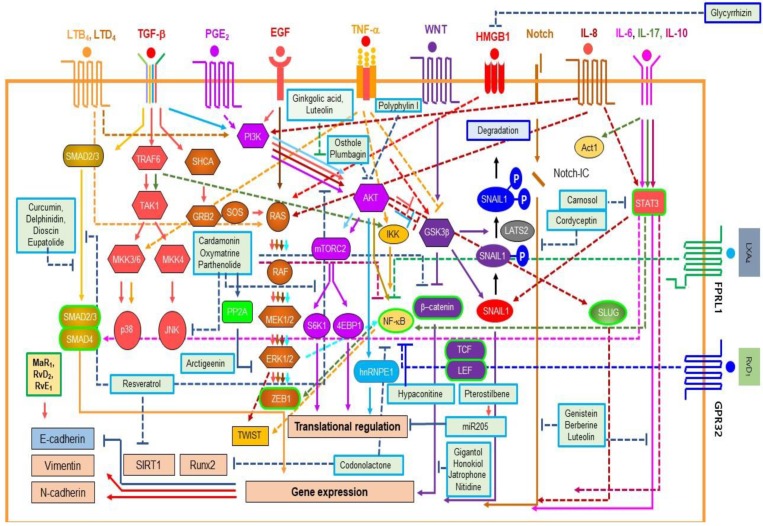
EMT inducers from the chronic tumor microenvironment and EMT repressors from natural anti-inflammatory compounds and pro-resolving lipids. EMT signaling process is simplified due to space limitation. Dot line (--) means indirect effects. Arrow (→) means promotion or induction. Bar line (--I) indicates suppression. Inbox demonstrates the Lats2-mediated snail1 degradation. Receptors here have several subtypes coupled to different signaling pathway. For instance, EP4 use the PI3K/AKT pathway. This figure only covers limited parts of EMT receptor signaling. Modified from Lee’s report [6]. 4EBP1: Eukaryotic Translation Initiation Factor 4E Binding Protein 1; Act1: NF-κB activator 1; Akt: AKR mouse thymoma; ERK: Extracellular Signal-Regulated Kinase; FPRL1: Formyl Peptide Receptor-Like 1; GSK3: Glycogen Synthase Kinase 3 Beta; GRB2: Growth Factor Receptor Bound Protein 2; hnRNPE2: Heterogeneous Nuclear Ribonucleoprotein E2; IKK: Inhibitor Of Nuclear Factor Kappa B Kinase; JNK: JUN N-Terminal Kinase; LATS2: Large Tumor Suppressor Kinase; LEF: Lymphoid Enhancer Binding Factor; MEK: MAPK/ERK Kinase; MKK: Mitogen-Activated Protein Kinase Kinase; mTORC: mammalian Target of Rapamycin Complex; NF-κB: Nuclear Factor Kappa B; PI3K: Phosphatidylinositol-4,5-Bisphosphate 3-Kinase; PP2A: Protein Phosphatase 2A; RAF: Rapidly Accelerated Fibrosarcoma; RAS: Rat Sarcoma Viral Oncogene homolog; Runx2: RUNX Family Transcription Factor 2; S6K1: Ribosomal protein S6 kinase beta-1; SHCA: Src Homology 2 Domain Containing Adaptor protein; SIRT: Sirtuin; SMAD: Sma- and Mad-Related Protein; SOS: Son of Sevenless; STAT3: Signal Transducer And Activator of Transcription 3; TAK1: TGF- Activated Kinase 1; TCF: Transcription Factor; TRAF6: TNF Receptor Associated Factor 6; TWIST: Twist Family BHLH Transcription Factor; ZEB1: Zinc Finger E-Box Binding Homeobox 1.

Here, we will briefly introduce EMT inducers from chronic inflammatory tumor microenvironment (Figure 3).

TNF-α is a critical determiner of inflammatory responses [57]. Serum concentration of TNF-α was determined as 1.47 pg/mL in invasive breast cancer patients and 0.98 ± 0.37 pg/mL in the control cohort [6,58]. TNF-α produced by macrophages can accelerate TGF- 1-induced EMT [59]. 

IL-6 serves as either a pro-inflammatory or anti-inflammatory cytokine [6]. The mean serum concentration of IL-6 was observed as 31.7 pg/mL in patients with breast cancer and 3.3 pg/mL in the normal cohort [6,60]. IL-6 induces EMT of human breast cancer cells [61]. IL-6/STAT3-induced expression of lncTCF7 can promote EMT of liver cancer cells [62]. The IL-6 pathway induces EMT in biliary tract cancer via cross-talking to the SMAD4 in the TGF- 1 pathway [63].

IL-8 is a chemokine mainly secreted by macrophages [64]. The mean serum concentration of IL-8 was found as 40.1 pg/mL in patients with breast cancer and 5.3 pg/mL in the normal group [60]. IL-8 expression is highly increased in TGF- 1-induced EMT in colon carcinoma and nasopharyngeal carcinoma [65]. IL-8 is also involved in mast cell-induced EMT of human lung and thyroid cancer cells [66,67]. JAK2/STAT3/Snail pathway is involved in the IL-8-induced EMT of HCC cells [68]. Brachyury-induced EMT of the tumor is mediated by IL-8/IL-8R signaling pathway [69].

IL-17 is a pro-inflammatory cytokine and mainly released from Th17 cells and macrophages [70]. IL-17 induces EMT of prostate cancers via MMP7 [71]. IL-17 induces EMT through STAT3 in the lung adenocarcinoma [72]. IL-17 can evoke self-renewal of CD133^+^ cancer cells in ovarian cancer [73].

High-mobility group box 1 (HMGB1) is a nuclear DNA-binding protein and released to the outside from macrophages, NK cells, dendritic cells, necrotic cells, and apoptotic cells according to infection, injury, and inflammation [74]. The mean serum HMGB1 level was 4.64 ng/mL in patients with malignant breast cancer, which was remarkably higher than in patients with benign breast cancer (1.32 ng/mL) or in healthy subjects (1.36 ng/mL) [75]. HMGB1 (2 μg/mL) induces EMT of colorectal and prostate cancer cells via the RAGE/NF-κB pathway [76,77].

IL-10 is a potent anti-inflammatory cytokine that suppresses T cell/macrophage cytokine synthesis and blocks their antigen-presenting capacity [78]. In vitro generated M1- and M2-macrophages both can induce EMT of pancreatic cancer cells via the IL-10 signaling pathway [79].

TGF- 1 is a potent anti-inflammatory cytokines [80]. Plasma TGF- 1 levels were significantly higher in stage IIIB/IV breast cancer patients (2.40 ng/mL) than those in healthy controls (1.30 ng/mL) [81]. It is a typical EMT inducer involved in cancer progression [6]. Please read other review for a detailed information about TGF- 1-induced EMT [6,82]. TGF- 1 promotes the production of IL-10 in macrophages from mouse cancer [83]. HMGB1 promotes expression of TGF- 1 via RAGE pathway to mediate TGF-1-induced EMT [84].

PGE2 is biosynthesized from arachidonic acids (Figure 2). The mean serum level of PGE2 was 6.324 pg/mL in patients with brain cancer and 1.677 pg/mL in the compared normal cohort [85]. PGE2 acts through G protein-coupled receptors such as EP1-EP4 [86]. PGE2 (2–10 μg/mL) works in an autocrine or paracrine manner, leading to stimulation of EMT through the expression of SNAIL and ZEB1 [87]. In turn, SNAIL induces blocking of degradation of PGE2 by repressing prostaglandin dehydrogenase, generating a positive loop that promotes cancer progression [88]. PGE2 (5 μM) enhances invasion of HCC cells via EP1-mediated expression of YB-1, which induces TGF- 1-induced EMT by AKT activation [89]. PGE2 (0.01–1 μM) inhibits fibroblast chemotaxis but stimulates chemotaxis of epithelial cells in the airway [90]. PGE2′s inhibitory action against TGF-1-induced EMT seems to be via the EP2 pathway since EP2 agonist can block TGF- 1-induced EMT [91]. As such, the effect of PGE2 on EMT varies depending on concentration and in cell types.

Leukotriene B4 (LTB4) can be produced via the 5-lipoxygenase pathway. Significantly higher levels of LTB4 have been found in the whole blood of lung cancer patients than those in the control group (44.1 vs. 17.9 pg/mL) [92]. LTB4 acts via two distinct GPCR called BLT1 and BLT2. BLT2 is involved in keratin phosphorylation and perinuclear reorganization, which is a prelude of EMT [93]. Accordingly, BLT2 is involved in the ras-promoted TGF-1-induced EMT [94]. As expected, LTB4 (100 nM) can induce EMT leading to vimentin expression through the BLT2/ERK2 activation [95].

Cysteinyl leukotrienes (CysLTs) include LTC4, LTD4, and LTE4 [96]. Mean serum levels of LTD4 found in HCC patients and healthy groups were 174.95 and 10.75 pg/mL, respectively [97]. Actions of CysLTs are mediated via GPCRs such as CysLT1 and CysLT2 [98]. LTD4 (100 nM) can suppress E-cadherin expression in cancer cells through enhanced translocation of -catenin to the nucleus while montelukast (0.1 mM) suppresses eosinophil-induced EMT in bronchial epithelial cells [99]. Recently, exosomes and cells from ascites in lung cancer patients can convert LTC4 to LTD4 to promote cancer cell migration and invasion via CysLT1 [100].

## 4. Reversal of EMT by Anti-inflammatory and Pro-Resolving Natural Compounds

The 2018 review by us did not cover the regulation of EMT by natural products [6]. In this part, we describe anti-inflammatory natural compounds and pro-resolving lipids that can prohibit EMT stimulated by mediators released in the tumor microenvironment.

### 4.1. Reversal of EMT by Anti-Inflammatory Natural Compounds

**Figure 4:**
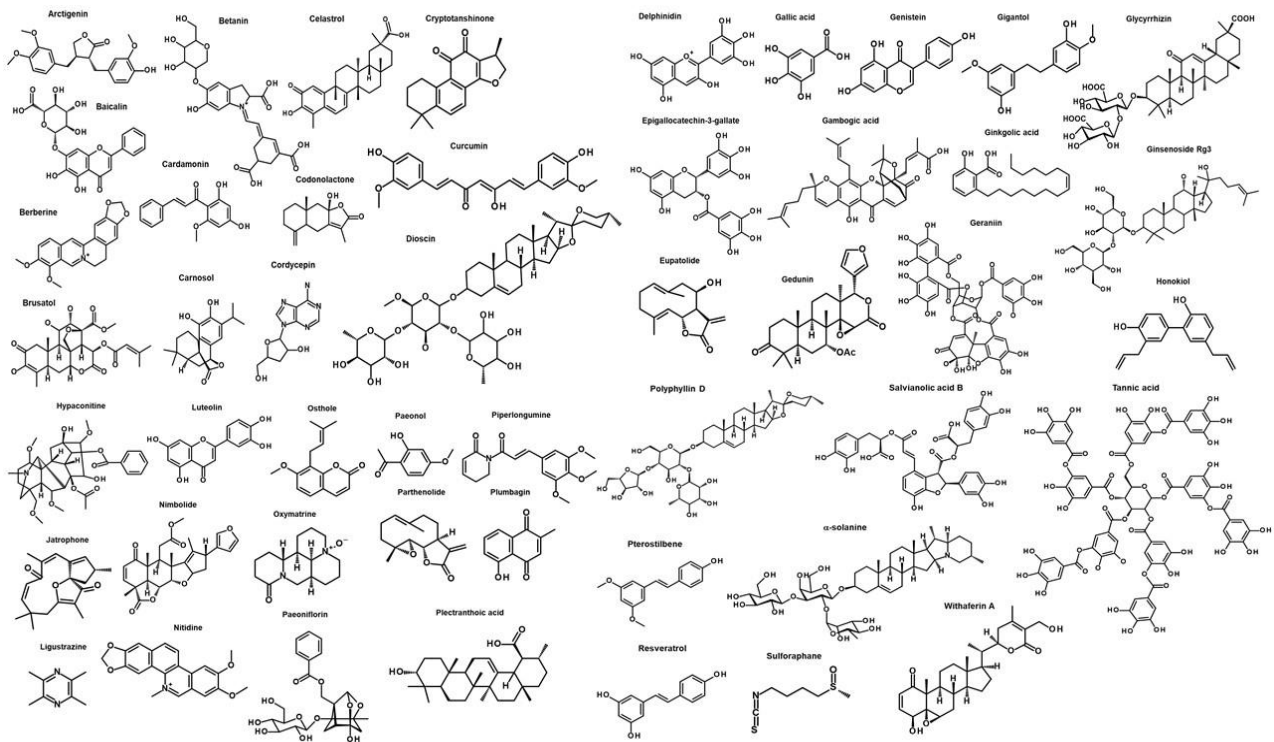
Structure of EMT inhibitors from natural sources.

Diverse natural products can inhibit EMT of cancer cells or epithelial cells. We focused on EMT inhibitors found from natural compounds that can inhibit the EMT of cancer cells caused by EMT inducers from the chronic inflammatory tumor microenvironment due to limited space (Figure 4, Table 1).

Arctigenin from Asteraceae has anti-inflammatory effects [101]. Arctigenin (12–50 μM) can suppress TGF--induced EMT of human lung cancer cells, thus blocking invasion [102]. Arctigenin (0.25–1 μM) can inhibit the expression of MCP-1 and subsequent EMT induced by ROS-dependent ERK/NF-κB pathway of renal tubular epithelial cells [103].

Baicalin found in *Scutellaria baicalensis* and *Scutellaria lateriflora* has well-known anti-inflammatory effects [104]. Baicalin (2 μM) can suppress TGF-1-mediated EMT in MCF10A cells by reducing the expression of slug [105]. Baicalin (12.5–25 μM) can inhibit the expression of TGF-1-induced EMT-related transcription factors in osteosarcoma cells and inhibit the aggressive metastasis of breast cancer by blocking EMT via inhibiting the activation of -catenin [106,107]

Berberine found in *Berberis* can reduce the secretion of IL-1 and TNF-α [108,109]. Berberine (5–20 μM) can reverse EMT in uterine cancer, leading to suppression of cancer metastasis [110]. Berberine can inhibit the metastatic ability of prostate cancer cells by suppressing EMT-associated genes [111]. Berberine (50 μM) can make nasopharyngeal carcinoma cells sensitive to radiation through EMT inhibition [112]. 

Betanin from beets can decrease the production of superoxide anion and cytokines TNF-α and IL-1 [113,114].

Betanin (25–50 μM) can inhibit high glucose-induced EMT of renal proximal tubular cells [115]. However, activity of betanin for EMT of cancer cells has not been reported yet.

Brusatol from the seeds of *Brucea sumatrana* can inhibit the response of cultured beta-cells to pro-inflammatory cytokines in vitro [116]. Brusatol (2 μM) can inhibit the EMT of pancreatic cancer cells [117].

Cardamonin, one of major component of *Alpinia katsumadai* has anti-tumor, anti-inflammatory, anti-nociceptive, and anti-itching activities [118,119,120]. Cardamonin (1–10 μM) can suppress TGF-1-stimulated EMT of A549 cells by restoring protein phosphatase 2A expression [120]. Cardamonin (5–20 μM) can block the invasiveness of human triple negative breast cancer cell by downregulation of Wnt/-catenin signaling pathway and induce the reversal of EMT [121]. Cardamonin (5–25 μM) also inhibits transglutaminase-2, one players in EMT, leading to JNK activation and NF-κB pathway [122].

Carnosol, found in *Rosmarinus officinalis* and *Salvia pachyphylla,* can block UV-induced inflammation through inhibition of STAT3 [123,124,125]. For a more in-depth look at various anti-inflammatory effects of carnosol, please refer to the 2017 review [126]. Carnosol (0.1–10 μM) controls the human glioblastoma stemness features by modulating EMT and inducing cancer stem cell apoptosis [127]. Carnosol (5–10 μM)-mediated SIRT1 activation inhibits the enhancer of zeste homolog 2 to attenuate liver fibrosis [128]. 

Celastrol (0.1–1 μM), from the root extracts of *Tripterygium wilfordii* and *Celastrus regelii*, can suppress experimental autoimmune encephalomyelitis [129]. Celastrol can inhibit the expression of snail and increased the expression of E-cadherin in the lung cancer cells [130]. Many studies have reported the effect of celastrol on EMT of diseases other than cancer (please refer the ref 128 by Kashyap et al.) [131].

Codonolactone, a major component of *Atractylodes lancea,* exhibits anti-allergic activity, anti-inflammatory, anticancer, gastroprotective, and neuroprotective activities [132,133]. Codonolactone (10–40 μM) can inhibit EMT in breast cancer cells by downregulating the transcriptional activity of Runx2 [134].

Cordycepin from the fungus *Cordyceps militaris* can suppress LPS-induced cytokine production by increasing heme oxygenase-1 expression [135,136]. Cordycepin (25–100 μM) can inhibit cancer stemness of TGF- induced chemo-resistant ovarian cancer cells [137]. Metronomic cordycepin therapy (25 mg/kg and 50 mg/kg) can prolong the survival of oral cancer-bearing mice and it (50 μM) inhibit EMT [138]. Cordycepin (100–200 μM) suppresses integrin/FAK signaling and EMT in HCC [139].

Cryptotanshinone, obtained from the root of *Salvia miltiorrhiza,* can protect against IL-1-induced inflammation in human osteoarthritis chondrocytes [140]. Cryptotanshinone (5–10 μM) targets tumor-initiating cells through down-regulation of stemness genes expression [141].

Curcumin, a phenolic compound found in *Zingiberaceae* turmeric, has strong anti-inflammatory, antioxidant, and antitumor properties [142]. A more extensive and detailed review of curcumin’s EMT has been reported recently [143]. Curcumin (25–50 μM) can inhibit metastasis in human papillary thyroid carcinoma cells by negatively regulating TGF-1-mediated Smad2/3 signaling pathway [142]. Curcumin (15 μM) can inhibit TNF-α-induced EMT in melanoma [144]. It can decrease EMT in cervical cancer cells by a pirin-dependent mechanism [145]. Pirin is a coregulatory of NF-κB involved in EMT [146]. Curcumin (30 μM) can suppress paraquat-induced EMT by blocking TGF- in A549 cells [147]. It (8 μM) can reverse oxaliplatin resistance caused by EMT in colorectal cancer through inhibition of the TGF-/Smad2/3 pathway [148].

Dioscin from roots of wild yam (*Dioscorea villosa*) shows potent anti-inflammatory effects via suppression of TNF-α-induced NF-κB-mediated VCAM-1, and ICAM-1 expression [149,150]. Dioscin (3 μM) can suppress TGF-1-induced EMT in A549 and HepG2 cells [149,151]. Dioscin (1–10 μM) also reverses HMT-induced EMT by down-regulating mdm2 and vimentin [152]. Diosgenin (400 μM), an aglycone of dioscin inhibits breast cancer cells with stem cell like properties by attenuation of the Wnt-catenin signaling [153].

Delphinidin, an anthocyanin, can reduce levels of inflammatory mediators including IL-6 and TNF-α induced by LPS [154]. Delphinidin (10–50 μM) inhibits TGF-1-induced EMT through a TGF- 1/Smad2 signaling pathway in glioblastoma cells and EGF-induced EMT in HCC cells [155,156].

Epigallocatechin-3-gallate found in green tea can regulates anti-inflammatory action through laminin receptor-mediated tollip signaling induction in LPS-stimulated human intestinal epithelial cells [157]. Epigallocatechin-3-gallate (25–50 μM) can also suppress nicotine-induced migration and invasion by blocking angiogenesis and EMT of non-small cell lung carcinoma (NSCLC) cells [158]. It (10–60 μM) also suppresses EMT and invasion in anaplastic thyroid carcinoma cells by blocking TGF-1/Smad pathways [159].

Eupatolide from the *Inula britannica* is used to treat bronchitis, disorders of the digestive system and inflammation [160]. Eupatolide can prohibit LPS-stimulated COX-2 and iNOS expression of RAW264.7 cells by evoking proteasomal degradation of TRAF6 [161]. Eupatolide (3–10 μM) can inhibit TGF-1-induced migration of breast cancer cells via down-regulation of SMAD3 phosphorylation and transcriptional repression of ALK5 [160].

Gallic acid is a trihydroxybenzoic acid found in gallnuts, sumac, witch hazel, and tea leaves [162]. For various anti-inflammatory effects of gallic acid, please refer to the 2017 review [163]. Gallic acid-coated sliver nanoparticle (50 μg/mL) can alter the expression of radiation-induced EMT in NSCLC [164]. Black tea polyphenols (10–40 μM) can also reverse EMT and inhibit invasion of human oral cancer cells [165].

Gambogic acid from the brownish or orange resin of *Garcinia hanburyi* can enhance the expression of heme oxygenase-1 through Nrf2 pathway and inhibit NF-κB and MAPK activation to mitigate inflammation in LPS-activated RAW264.7 cells [166]. Gambogic acid (0.5–1 μM) can suppress cancer invasion and migration by inhibiting TGF-1-induced EMT [167]. It (2 μM) can induce cleavage of vimentin in HeLa cells [168].

Gedunin, one of the main chemical compounds in the neem tree, can protect TLR-mediated inflammation by suppression of inflammasome activation and cytokine production [169,170]. Gedunin (15 μM) suppresses EMT of pancreatic cancer by inhibiting sonic hedgehog signaling pathway [171]. 

Genistein first isolated from *Genista tinctoria* can suppress psoriasis-related inflammation through a STAT3/NF-κB-dependent mechanism in keratinocytes [172,173]. For more detailed information about genistein’s anti-inflammatory action or anti-EMT, please refer to a previous review by Spagnulo et al. and Lee et al. [174,175]. Genistein (200 μM) can induce apoptosis of colon cancer cells by reversal of EMT via a notch1/NF-κB/slug/E-cadherin pathway [176]. miR-223 inhibitor and genistein (20 μM) can synergistically reverse in EMT of gemcitabine-resistant pancreatic cancer cells [177]. 

Geraniin found in geraniums can ameliorate experimental acute reflux esophagitis via NF-κB-regulated anti-inflammatory activities in rats [178,179]. It (15–20 μM) can also suppress TGF-1-induced EMT, migration, invasion and anoikis resistance in A549 lung cancer cells [180].

Gigantol from the *Cymbidium goeringii* can suppress LPS-stimulated iNOS and COX-2 expression through NF-κB inactivation in RAW 264.7 cells [181]. It (5–20 μM) can attenuate cancer stem cell-like phenotypes and induce anoikis in human lung cancer H460 cells [182,183].

Ginkgolic acid from *Ginkgo biloba* can significantly inhibit the production of NO, PGE_2_, and pro-inflammatory cytokines in ox-LDL-stimulated HUVECs cells [184]

Ginkgolic acid (100 μM) can inhibit TGF-1-induced EMT of lung cancer cells through PI3K/AKT/mTOR inactivation [185]. PPAR-γ might be involved in the suppression of EMT since ginkgolic acid is a PPAR-γ modulator.

Ginsenosides Rh1, Rg3, Rb1, Rg5, and Rg1 from ginseng can block inflammatory responses by inhibiting the activation of NLRP3, NLRP1, and AIM [186]. Rg3 (25–100 μM) can suppress EMT and invasion in lung cancer cells by reducing expression of FUT4 [187]. It (75 μg/mL) can also sensitize hypoxic lung cancer cells to cisplatin via blocking of NF-κB mediated EMT [188]. Rb1 (160 μg/mL) can inhibit hypoxia-induced EMT in ovarian cancer cells by regulating miR-25 [189]. Downregulation of HDAC3 by Rg3 (25 and 50 μg/mL) can inhibit EMT of cutaneous squamous cell carcinoma through c-Jun acetylation [190]. Rg3 (75 μg/mL) can inhibit growth and EMT of human oral squamous carcinoma cells by down-regulating miR-221 [191]. 

Glycyrrhizin (50–200 μM) from the roots of *Glycyrrhiza glabra* (Licorice) has anti-inflammatory and antiviral activities. It is also a novel pharmacological inhibitor of HMGB1 [192]. Glycyrrhizin can attenuate the EMT of prostate cancer cells by suppressing HMGB1-involved signaling pathway [192].

Honokiol from the *Magnolia* possesses anti-inflammatory activity by blocking downstream signaling of MEKK-1 in NF-κB activation pathway [193,194]. It (30 μM) can also inhibit EMT-mediated migration of human NSCLC cells in vitro by targeting c-FLIP and EMT of breast cancer cells by targeting STAT3/Zeb1/E-cadherin axis [195,196]. Honokiol (20 μM) can inhibit the metastasis of renal cancer cells by blocking EMT through regulating miR-141/ZEB2 pathway [197]. It (5–20 μM) can also inhibit the invasion of U87MG human glioblastoma cell via regulation of EMT [198]. 

Hypaconitine from the root of *Aconitum* species can suppress 0.1% histamine-induced acute inflammation without showing an ulcerogenic effect [199]. Hypaconitine (8 μM) can inhibit TGF-1-evoked EMT of A549 lung cancer cells possibly by blocking NF-κB activation [200]. 

Jatrophone from *Jatropha isabellei* has anti-nociceptive and anti-inflammatory activities [201]. Jatrophone (2 μM) can interfere with Wnt/-catenin signaling and reverses EMT of human triple-negative breast cancer [202].

Ligustrazine found in nattō and in fermented cocoa beans can significantly decrease CCL3, CCL19, CCl21, IL-4, IL-5, and IL-17A in bronchoalveolar lavage fluid of ovalalbumin-induced mice [203,204]. There are many reports of anti-inflammatory effects of ligustrazine [205]. Ligustrazine (100 μM) can suppress EMT of renal cell carcinoma cells by blocking MMP9 and TGF-1 [206].

Luteolin is a natural flavonoid that possesses anti-inflammatory and anti-cancer activities [207]. Luteolin (40 μM) inhibits TGF-1-induced EMT of A549 lung cancer cells through interfering with PI3K/AKT/NF-κB/Snail pathway [207]. Luteolin (10 μM) can suppress EMT and negatively regulating -catenin expression in breast cancer cells [208,209]. It (5–20 μM) can inhibit metastasis of melanoma cells by decreasing HIF-1α/VEGF signaling-mediated EMT [210]. It (5 μM) can also inhibit EMT of colorectal cancer cell by suppressing CREB1 expression [211]. Luteolin (20 μM) can inhibit the invasion of cervical cancer by blocking EMT signaling [212]. It (30 μM) can block gastric cancer progression by reversing EMT through inhibition of the notch signaling [213]. It (15.6–31.3 μM) can also inhibit EMT in paclitaxel-resistant ovarian cancer cells [214]. Luteolin (20–160 μM) can also block IL-6-induced EMT in pancreatic cancer cells by inhibiting STAT3 signaling [215].

Nimbolide, from the neem tree (*Azadirachta indica*), can inhibit NF-κB pathway in intestinal epithelial cells and macrophages, resulting in alleviation of experimental colitis in mice [216]. Nimbolide (5 μM) can suppress pancreatic cancer growth and metastasis through inhibition of EMT [217]. It (1–6 μM) can also suppress NSCLC cell invasion and migration via manipulation of DUSP4 expression and ERK1/2 signaling [218].

Nitidine chloride, a pentacyclic alkaloid isolated from the root of *Zanthoxylum nitidium*, can suppress LPS-induced interleukin production via MAPK and NF-κB in RAW 264.7 cells [219]. Hedgehog pathway is implicated in nitidine chloride (2.5 μM)-induced blocking of EMT of breast cancer cells [220]. Nitidine chloride (5 μM) can also inhibit EMT of osteosarcoma cell via Akt/GSK-3/snail signaling pathway [221].

Osthole, the major natural coumarin from *Cnidium monnieri* (L.) Cuss, exerts anti-inflammatory effects by blocking of the activation of the NF-κB and MAPK/p38 pathways [222]. Osthole (20 μM) can suppress HGF-induced EMT via repression of the c-Met/Akt/mTOR pathway in human breast cancer cells [223]. Osthole (20–40 μM) can also inhibit IGF-1-induced EMT by inhibiting PI3K/Akt signaling pathway in human brain cancer cells [224]. By inhibiting snail signaling and miR-23a-3p, osthole (20–80 μM) can suppress EMT-mediated metastatic ability in prostate cancer [225]. Osthole (5–20 μM) an also inhibit TGF -induced EMT by suppressing NF-κB mediated snail activation in A549 cells [226].

Oxymatrine, the active component from Radix *Sophorae flavescentis*, is well known for its anti-inflammatory activity [227]. Oxymatrine (1.5–6 μM) can reverse EMT of breast cancer cells by depressing αⅤ3 integrin/FAK/PI3K/Akt signaling activation [228]. It (0.25–0.75 μM) can inhibit EMT of colorectal cancer cells by suppressing NF-κB signaling [229]. Chronic oxymatrine treatment can induce resistance and EMT of colorectal cancer cells [230].

Paeoniflorin from *Paeonia lactiflora* has anti-inflammatory effects [231,232]. Paeoniflorin (5–10 μM) can suppress EMT of human colorectal cancer cells and glioblastoma cells and prevent hypoxia-induced EMT of human breast cancer cells [233,234].

Paeonol found in *Paeonia suffruticosa* (moutan cortex) can suppress LPS-induced HMGB1 translocation from the nucleus to the cytoplasm in RAW264.7 cells [235,236]. It (60–120 μM) can attenuate aging of MRC-5 cells and inhibit EMT of HaCaT cells induced by aging MRC-5 cell-conditioned medium [237].

Parthenolide from *Tanacetum parthenium* has well-known anti-inflammatory activities [238]. Parthenolide (5 μM) can inhibit TGF-1-induced EMT of colorectal cancer cells [239]. Parthenolide (10–20 μM) can suppress HIF-1α signaling and hypoxia-induced EMT in colorectal cancer [240]. Parthenolide binds Gly-Leu-Ser/Lys- “co-adaptation pocket” to inhibit EMT of lung cancer cell [241].

Plectranthoic acid isolated from *Ficus microcarpa*, can alleviate the symptoms of type 2 diabetes mellitus by inhibiting dipeptidyl peptidase 4 [242]. Plectranthoic acid is a novel activator of AMPK can induce apoptotic death in prostate cancer cells [243]. Plectranthoic acid (20–40 μM) can suppress EMT of prostate cancer [244].

Piperlongumine, a constituent of the fruit of the long pepper (*Piper longum*) can inhibit neuroinflammation via regulating NF-κB signaling pathways in LPS-stimulated BV2 microglia cells [245,246]. Piperlongumine (1–5 μM) inhibits TGF--induced EMT by modulating the expression of E-cadherin, Snail1, and Twist1 [247]. 

Plumbargin from *Plumbago* genus can attenuate the expression of inflammatory cytokine in LPS-activated BV-2 cells [248]. Plumbagin (0.5–1 μM) can inhibit EMT of human cervical carcinoma cells and inhibit tumor invasion of endocrine-resistant breast cancer through EMT [249]. It (0.1–0.5 μM) suppresses EMT via inhibiting Nrf2-mediated signaling pathway in human tongue squamous cell carcinoma cells [250]. Plumbagin (1–5 μM) can inhibit PI3K/Akt/mTOR-mediated EMT in human pancreatic cancer cells [251]. Plumbagin (1–5 μM) shows differential proteomic responses to EMT of PC-3 and DU145 prostate cancer cells [252].

Polyphyllin I, a component in the Rhizoma of Paris, can improve collagen-induced arthritis by blocking the inflammation response in macrophages through the NF-κB Pathway [253]. Polyphyllin I (0.3 μM) can overcome EMT-associated resistance to erlotinib in lung cancer cells via IL-6/STAT3 pathway inhibition [254].

Pterostilbene (5–10 μM) from blueberries can effectively suppress the generation of cancer stem cells and metastatic potential under the influence of M2 TAMs by modulating EMT associated signaling pathways, specifically the NF-κB/miR488 circuit [255]. Pterostilbene (10 μM) can also inhibit triple-negative breast cancer metastasis by inducing miR205 expression and negatively modulates EMT [256]. Long non-coding RNAs such as MEG3, TUG1, H19, and DICER1-AS1 contribute to the inhibitory effect of pterostilbene (1–50 μM) on proliferation and EMT of human breast cancer cells [257].

Resveratrol, a constituent of grapes and various other plants, is an activator of PPAR and SIRT1 [258]. Resveratrol (12 μM) can suppress TGF-1-induced EMT in colorectal cancer through the TGF-1/SMADs signaling pathway [259]. Resveratrol (20 μM) can inhibit TGF-1-induced EMT and suppress lung cancer invasion and metastasis [260]. Resveratrol (50 μM) can inhibit the EMT of pancreatic cancer cells through suppression of the PI3K/Akt/NF-κB pathway [261]. Resveratrol might inhibit EMT via downregulation of COX and SIRT1 activation, although roles of SIRT1 in EMT of cancer cells show conflicting results [262,263].

Salvianolic acid, an active compound present in *Salvia miltiorrhiza,* can suppress CCL-20 expression in TNF-α-treated macrophages [264]. Salvianolic acid B (1–100 μM)-induced expression of miR-106b-25 can suppress EMT of HK-2 cells [265]. Salvianolic acid B (1–10 μM) can prevent EMT through the TGF-1 signal transduction pathway [266].

α−Solanine is a glycoalkaloid poison found in species of the nightshade family within the genus *Solanum*, such as the potato and the eggplant [267]. A chloroform fraction of *Solanum nigrum* can suppresses nitric oxide and TNF-α in LPS-stimulated mouse peritoneal macrophages through inhibition of p38, JNK and ERK1/2 [268]. α-Solanine (4–12 μM) can suppress the invasion of human prostate cancer cell by inhibiting EMT and MMPs expression [269].

Sulforaphane from cruciferous vegetables may directly impair the formation of NLRP3 inflammasome by inhibiting ASC or caspase-1 [270]. Sulforaphane (1–5 μM) can also inhibit the EMT and metastasis of human lung cancer through miR616-5p-involved GSK3/-catenin pathways [271]. Sulforaphane (20–40 μM) can suppress TGF-1-induced EMT of HCC cells via the ROS-dependent pathway [272]. It (5–20 μM) can block the EMT of human bladder cancer cells via COX-2/MMP2,9/SNAIL, ZEB1, and miR200c/ZEB1 pathways [273].

Tannic acid is a type of polyphenol inhibits NLRP3 inflammasome-mediated IL-1 production via blocking NF-κB signaling in macrophages [274]. Tannic acid (25 μM) attenuates TGF-1-induced EMT in lung epithelial cells [275].

Withaferin A from the Solanaceae family attenuates bleomycin-induced scleroderma by targeting FoxO3a and NF-κB signaling [276]. Withaferin A (0.5 μM) can inhibit the EMT of NSCLC cells [277]. It (2 μM) can also inhibit the EMT of MCF10A cells and suppress vimentin expression in breast tumors [278]. Extracts of root in *Withania somnifera* can suppress mammary EMT and cancer metastasis [279].

### 4.2. Reversal of EMT by Natural Pro-resolving Lipids 

Pro-resolving lipids can be useful agent against cancer and EMT of cancer has been studied since cancer is understood as a non-resolving disease [6,280]

Here, we briefly introduced the effect of pro-resolving lipids on EMT of cancer cells and emphasized natural sources of pro-resolving lipids (Table 2).

Pro-resolving lipids are generally known to act via GPCR receptors such as BLT1, CMKLR1, FPRL1 (ALX/FPR2), GPR18, and GPR3 [41,281]. CMKLR1 is a receptor with high affinity for RvE1, as measured via radioligand-binding assay (Kd = 11.3–5.4 nM) [282,283]. BLT1 is also a receptor with low affinity for RvE1, as measured via radioligand-binding assay (Kd = 45 nM) [284]. CMKLR1 has not been reported to be associated with EMT in cancer cells but seems to be associated with EMT in diabetic nephropathy [285]. FPRL1 (ALX/FPR2) not only possesses an annexin A1 [286] protein but also LxA4 (Kd = 1.7 nM, radioligand binding) [287], AT-RvD1 (EC50 = 1.8 × 10^−10^, -arrestin receptor system [288], RvD1 (Kd = 0.17 ± 0.06 nM, radioligand assay; EC50 = 4.5 × 10–11, -arrestin receptor system) [288], RvD3 (activation of FPRL1 at 100 nM) [289] and other similar proteins as a ligand. FPRL1 mediates EMT inhibition by LxA4 and RvD1 [290,291].

GRP18 has been reported as a receptor for RvD2 on performing GPCR--arrestin-based screening (Kd of 9.6 ± 0.9 nM, radioligand binding) [292]. GPR32 is known as the receptor of RvD1 (EC50 = 3.6 × 10^−12^, -arrestin receptor system) [293] and RvD5 (activation of GPR32 at the range of 10^−13^–10^−9^ M) [294]. GPR32 is also activated by RvD3 and AT-RvD3 (0.1 pM–10 nM) [295]; it mediates EMT Inhibition by RvD1 [291]. RvD1 also promotes wound healing in pulmonary epithelial cells and mediates EMT inhibition [296].

LxA4 (0.1–0.8 μM) can suppress TGF-1 signaling in pancreatic cancer cells, reverse mesenchymal features and block invasion and migration via a FPR2 [297]. LxA4 (0.01–0.1 μM) can also suppress estrogen-induced EMT via LxA4 receptor-dependent manner in endometriosis [298]. LxA4 (0.2 μM) and its analogue can suppress EMT, migration and metastasis of HCC by regulating integrin-linked kinase axis [299].

100 nM of RvD1 and RvD2 can inhibit TGF-1-induced EMT of A549 lung cancer cells through FPR2/ALXR and GPR32 [291]. GPR32 can recognize RvD1 (EC_50_ = 8.8 pM from -arrestin receptor system) as an endogenous ligand [293]. Aspirin-triggered RvD1 (10 ng/mL) can block TGF-1-induced EMT of A549 lung cancer cells via suppression of the mTOR pathway by reducing the expression of pyruvate kinase M2 [300]. RvD1 (400 nM) prevents EMT of HCC cells by inhibiting paracrine of cancer-associated fibroblast-derived cartilage oligomeric matrix protein [301].

MaR1 (0.1–10 ng/mice), a docosahexaenoic acid-derived pro-resolution lipid, can protect skin from inflammation and oxidative stress caused by UVB irradiation [302]. Receptor for MaR1 is yet unknown. The effect of MaR1 on EMT of cancer cells has not been reported yet except one study has shown that incubating DHA with A549 lung cancer cells can produce MaR1 (1.58 ng/mL) and PD1 (1.67 ng/mL) [303]. MaR1 (10 nM) can inhibit TGF-1-induced proliferation, migration, and differentiation in human lung fibroblasts [304].

PD1 has anti-inflammatory and survival effects on neuronal diseases such as Alzheimer’s disease and retinal degenerations [305]. PDX (1–100 nM), one of PD1 derivatives, can suppress bleomycin-induced lung fibrosis through blocking EMT [6,306]. However, it is hard to find reports about the effect of PDs on the EMT of cancer cells [6,306]. However, it is hard to find reports about the effect of PDs on the EMT of cancer cells.

Pro-resolving lipid classes are mainly originated from ω-3 fatty acids which constitute a group of essential fats that humans cannot synthesize endogenously [307]. Several pro-resolving lipids can be obtained by total synthesis. Studies on derivatives are in progress.

It is possible to produce pro-resolving lipids by biological methods. EPA and DHA are the major long chain ω-3 fatty acids in the diet. Algae are the major producers of EPA and DHA in the ecosystem. Therefore, fish that consumes algae contains a lot of EPA and DHA [307]. Accordingly, algae or fish might be a starting point for isolating or producing pro-resolving lipids in industrial scale. Brain cells of rainbow trout (*Oncorhynchus mykiss*) can produce novel DHA-derived Rvs and PDs (Table 2) [308]. However, baking reduces proportions of PG, Rv, and hydroxy-fatty acid in farm-raised Atlantic salmon (*Salmo salar*; Table 2) [309].

Infectious organisms can produce pro-resolving lipids to control host inflammation. Thus, supraphysiological levels of LxA4 are generated during infection by *Toxoplasma gondii*, which in turn reduces IL-12 production by dendritic cells, thus dampening Th1-type cell-mediated immune responses (Table 2) [310].

*Candida albicans* can modulate host defense by biosynthesizing the pro-resolving lipid RvE1 [311]. *C. albicans* can biosynthesize nanogram quantities of RvE1 from EPA without collaboration of other cellular partners. It can also biosynthesize PDs (Table 2) [311]. *Trypanosoma cruzi* is a protozoan parasite that causes Chagas disease and produces the RvD1, RvD5, and RvE2 (Table 2) [312]. These reports suggested that algae, fish, and some infectious organisms might be applied to produce pro-resolving lipids.

## 5. Perspectives

Several natural compounds have anti-inflammatory activities and/or anti-EMT activities. However, few reports have explained their anti-EMT activity by anti-inflammatory or pro-resolving mechanisms. Therefore, examining anti-EMT activities of natural compounds based on their anti-inflammatory or pro-resolving activities and assuring their anti-EMT activities in vivo might be important in the future. Especially, it might be a reasonable way to study the anti-EMT activity of natural products through interaction with the tumor microenvironment [255].

The flux of inflammatory or pro-resolving lipids from arachidonic acid related pathway (Figure 2) could change if one pathway is blocked by natural anti-inflammatory or pro-resolving compounds. Therefore, prevention of the production of these lipids might influence levels of other inflammatory and pro-resolving lipids. Thus, further studies on effects of anti-inflammatory compounds or pro-resolving lipids on EMT of cancer might require entire profiles of lipid metabolites affecting the EMT.

## 6. Conclusions

Expansion of studies about other pro-resolving lipids and nature-derived pro-resolving compounds to inhibit EMT are needed. To do this, various types of pro-resolving lipids and new compounds from natural sources should be procured and diffused to researcher without barrier. Thus, collaboration of natural chemistry researchers with other fields is also required.

## Figures and Tables

**Table 1:** Lists of EMT inhibitors from natural sources.

Name	Source	Name	Source
Arctigenin	*Astraceae*	Glycyrrhizin	*Glycyrrhiza glabra*
Baicalin	*Scutellaria baicalensis, Scutellaria lateriflora*	Honokiol	*Magnolia*
Berberine	*Berberis*	Hypaconitine	*Aconitum*
Betanin	beets	Jatrophone	*Jatropha isabellei*
Brusatol	*Brucea sumatrana*	Ligustrazine	Nattō, fermented cocoa beans
Cardamonin	*Alpinia katsumadai*	Luteolin	*Reseda luteola*
Carnosol	*Rosmarinus officinalis, Salvia pachyphylla*	Nimbolide	*Azadirachta indica*
Celastrol	*Tripterygium wilfordii, Celastrus regelii*	Nitidine chloride	*Zanthoxylum nitidium*
Codonolactone	*Atractylodes lancea*	Osthole	*Cnidium monnieri*
Cordycepin	*Cordyceps militaris*	Oxymatrine	*Sophorae flavescentis*
Cryptotanshinone	*Salvia miltiorrhiza*	Paeoniflorin	*Paeonia lactiflora*
Curcumin	*Zingiberaceae*	Paeonol	*Paeonia suffruticosa*
Dioscin	*Dioscorea villosa*	Parthenolide	*Tanacetum parthenium*
Delphinidin	*Viola, Delphinium*	Plectranthoic acid	*Ficus microcarpa*
Epigallocatechin-3-gallate	Green tea	Piperlongumine	*Piper longum*
Eupatolide	*Inula britannica*	Plumbargin	*Plumbago*
Galic acid	gallnuts, sumac, witch hazel, tea leaves	Polyphyllin I	Rhizoma of Paris
Gambogic acid	*Garcinia hanburyi*	Pterostilbene	blueberries
Gedunin	*Azadirachta indica*	Resveratrol	grapes
Genistein	*Genista tinctoria*	Salvianolic acid	*Salvia miltiorrhiza*
Geraniin	Geraniums	α−Solanine	*Solanum*
Gigantol	*Cymbidium goeringii*	Sulforaphane	cruciferous vegetables
Ginkgolic acid	*Ginkgo biloba*	Tannic acid	*Caesalpinia spinosa*
Ginsenosides	Ginseng	Withaferin A	Solanaceae

**Table 2:** Pro-resolving lipids. * Antagonist.

Name	Formula	Receptor	Anti-EMT	Source	Yields (pg/mg)
*AA-derived lipoxins*					
LxA4	5S,6R,15S-trihydroxy-7E,9E,11Z,13E-ETE	FPR2, GPR32	+	*Toxoplasma gondii*	~0.43 ng
LxB4	5S,14R,15S-trihydroxy-6E,8Z,10E,12E-ETE				
AT-LxA4	5S,6R,15R-trihydroxy-7E,9E,11Z,13E-eicosatetraenoic acid	FPR2			
AT-LxB4	5S,14R,15R-trihydroxy-6E,8Z,10E,12E-eicosatrienoic acid				
*EPA-derived resolvins*					
RvE1	5S,12R,18R-trihydroxy-6Z,8E,10E,14Z,16E-EPA	CMKLR1, BLT *, TRPV1 *,	+ (?)	*Candida albicans*	
18S-RvE1	5S,12R,18S-trihydroxy-6Z,8E,10E,14Z,16E-EPA	CMKLR1, BLT *			
RvE2	5S,18R-dihydroxy-6E,8Z,11Z,14Z,16E-EPA	CMKLR1, BLT *		*Trypanosoma cruzi*	9.5–23.6
RvE3	17R,18R/S-dihydroxy-5Z,8Z,11Z,13E,15E-EPA				
*DHA-derived resolvins*					
RvD1	7S,8R,17S-trihydroxy-4Z,9E,11E,13Z,15E,19Z-DHA	GPR32, FPR2, TRPV3 *,	+	*Trypanosoma cruzi, Oncorhynchus mykiss*	1.8–7.0
RvD2	7S,16R,17S-trihydroxy-4Z,8E,10Z,12E,14E,19Z-DHA	GPR32, GPR18, FPR2,	+	*Oncorhynchus mykiss*	
RvD3	4S,11R,17S-trihydroxy-5Z,7E,9E,13Z,15E,19Z-DHA	GPR32			
RvD4	4S,5R,17S-trihydroxy-6E,8E,10Z,13Z,15E,19Z-DHA				
RvD5	7S,17S-dihydroxy-4Z,8E,10Z,13Z,15E,19Z-DHA	GPR32		*Trypanosoma cruzi;* *Oncorhynchus mykiss*	
RvD6	4S,17S-dihydroxy-5E,7Z,10Z,13Z,15E,19Z-DHA				
AT-RvD1	7S,8R,17R-trihydroxy-4Z,9E,11E,13Z,15E,19Z-DHA	FPR2, GPR32, TRPV3 *,	+		
AT-RvD2	7S,16R,17R-trihydroxy-4Z,8E,10Z,12E,14E,19Z-DHA				
AT-RvD3	4S,11R,17R-trihydroxy-5Z,7E,9E,13Z,15E,19Z-DHA	GPR32			
AT-RvD4	4S,5R,17R-trihydroxy-6E,8E,10Z,13Z,15E,19ZDHA				
AT-RvD5	7S,17R-dihydroxy-4Z,8E,10Z,13Z,15E,19Z-DHA				
AT-RvD6	4S,17R-dihydroxy-5E,7Z,10Z,13Z,15E,19Z-DHA				
*n-3 DPA-derived resolvins*					
RvT1	7,13R,20-trihydroxy-8E,10Z,14E,16Z,18E-DPA				
RvT2	7,8,13R-trihydroxy-9E,11E,14E,16Z,19Z-DPA				
RvT3	7,12,13R-trihydroxy-8Z,10E,14E,16Z,19Z-DPA				
RvT4	7,13R-dihydroxy-8E,10Z,14E,16Z,19Z-DPA				
RvD1n-3	7,8,17-trihydroxy-8,10,13,15,19-DPA				
RvD2n-3	7,16,17-trihydroxy-8,10,12,14,19-DPA				
RvD5n-3	7,17-dihydroxy-8,10,13,15,19-DPA				
*DHA-derived protectins/neuroprotectins*					
PD1(NPD1)	10R,17S-dihydroxy-4Z,7Z,11E,13E,15Z,19Z-DHA			*Oncorhynchus mykiss*	
PDX	10S,17S-dihydroxy-4Z,7Z,11E,13Z,15E,19Z-DHA		+ (?)		
22-hydroxy-PD1	10R,17S,22-trihydroxy-4Z,7Z,11E,13E,15Z,19Z-DHA				
AT-PD1	10R,17R-dihydroxy-4Z,7Z,11E,13E,15Z,19Z-DHA				
Ent-AT-NPD1	10S,17S-Dihydroxy-4Z,7Z,11E,13E,15Z,19Z-DHA				
*n-3 DPA-derived protectins/neuroprotectins*					
PD1n-3	10,17-dihydroxy-7,11,13,15,19-DPA				
PD2n-3	16,17-dihydroxy-7,10,12,14,19-DPA				
*DHA-derived maresins*					
MaR1	7R,14S-dihydroxy-4Z,8E,10E,12Z,16Z,19Z-DHA	TRPV1 *, TRPA1 *	+ (?)		
MaR2	13R,14S-dihydroxy-4Z,7Z,9E,11E,16Z,19Z-DHA				
7-epi-MaR1	7S,14S-dihydroxy-4Z,8E,10Z,12E,16Z,19Z-DHA				
MaR-L1	14S,22-dihydroxy-4Z,7Z,10Z,12E,16Z,19Z-DHA				
MaR-L2	14R,22-dihydroxy-4Z,7Z,10Z,12E,16Z,19Z-DHA				
*n-3 DPA-derived maresins*					
MaR1n-3	7S,14S-dihydroxy-8E,10E,12Z,16Z,19Z-DPA				
MaR2n-3	13,14-dihydroxy-7Z,9,11,16Z,19Z-DPA				
MaR3n-3	7,14-dihydroxy-8,10,12,16Z,19Z-DPA				

* antagonist; +(?) confirmed in non-cancer cells.
